# Qin Huang formula enhances the effect of Adriamycin in B-cell lymphoma via increasing tumor infiltrating lymphocytes by targeting toll-like receptor signaling pathway

**DOI:** 10.1186/s12906-022-03660-8

**Published:** 2022-07-11

**Authors:** Weili Li, Lingling Lv, Ming Ruan, Jiayue Xu, Wenhua Zhu, Qiong Li, Xufeng Jiang, Lan Zheng, Weirong Zhu

**Affiliations:** 1 Department of Traditional Chinese Medicine, Ruijin Hospital, Shanghai Jiao Tong University School of Medicine, Shanghai, 200025 China; 2 Department of Nuclear Medicine, Ruijin Hospital, Shanghai Jiao Tong University School of Medicine, Shanghai, 200025 China

**Keywords:** Qin Huang formula, B-cell lymphoma, Tumor microenvironment, Tumor-infiltrating lymphocytes, Toll-like receptor, Systemic pharmacology

## Abstract

**Background:**

As an original traditional Chinese medicinal formula, Qin Huang formula (QHF) is used as adjuvant therapy for treating lymphoma in our hospital and has proven efficacy when combined with chemotherapy. However, the underlying mechanisms of QHF have not been elucidated.

**Methods:**

A network pharmacological-based analysis method was used to screen the active components and predict the potential mechanisms of QHF in treating B cell lymphoma. Then, a murine model was built to verify the antitumor effect of QHF combined with Adriamycin (ADM) in vivo. Finally, IHC, ELISA, ^18^F-FDG PET-CT scan, and western blot were processed to reveal the intriguing mechanism of QHF in treating B cell lymphoma.

**Results:**

The systemic pharmacological study revealed that QHF took effect following a multiple-target and multiple-pathway pattern in the human body. In vivo study showed that combination therapy with QHF and ADM potently inhibited the growth of B cell lymphoma in a syngeneic murine model, and significantly increased the proportion of tumor infiltrating CD4+ and CD8+ T cells in the tumor microenvironment (TME). Furthermore, the level of CXCL10 and IL-6 was significantly increased in the combination group. Finally, the western blot exhibited that the level of TLR2 and p38 MAPK increased in the combination therapy group.

**Conclusion:**

QHF in combination of ADM enhances the antitumor effect of ADM via modulating tumor immune microenvironment and can be a combination therapeutic strategy for B cell lymphoma patients.

**Supplementary Information:**

The online version contains supplementary material available at 10.1186/s12906-022-03660-8.

## Introduction

Non-Hodgkin lymphoma (NHL) is a malignancy that mainly infringes the lymphoid system, with extranodal sites often affected as well. It has arisen to the top 10 most common cancers in 2020 [1]. Among all the subtypes of NHLs, diffuse large B cell lymphoma (DLBCL) is the most common subtype and accounts for 25-30% of diagnoses [2]. Albeit aggressive, most DLBCL is chemo-sensitive, and R-CHOP (rituximab, cyclophosphamide, doxorubicin, vincristine, and prednisone) is the standard regimen. However, due to resistance to R-CHOP, there are still ~ 30% of patients suffering from refractory or relapse (r/r) disease, and their prognosis is rather poor [3, 4]. Such treatment plight has prompted the emergence of novel treatment options, such as therapeutics targeting immune checkpoints, tumor microenvironment (TME), signaling pathways, and cellular immunotherapy [5]. As a cellular immunotherapy, chimeric antigen receptor (CAR) T-cell therapy has shown a promising future in the management of B cell lymphomas. Two CAR T-cell products are now approved for the treatment of relapsed/refractory large B-cell lymphoma [6]. However, its acute and late toxicities, as well as on-target off-tumor effect should be addressed [7]. PD-1/PD-L1 blockade, as an immune checkpoint inhibitor (ICI), aims to protect cytotoxic T cells and restore their function from PD-1/PD-L1 mediated immune evasion. However, its efficacy in DLBCL remains unsatisfactory compared to classical Hodgkin Lymphoma (cHL) [8, 9]. Research accumulated that the pre-existing immune landscape within the TME may influence the response to immunotherapies [10–12] and R-CHOP [13, 14]. Therefore, it is pivotal to explore agents that can modulate TME. TME is an extremely intricate environment that harbors diverse cells, structures, and matters, with one component interacting with many others, forming complex regulatory networks. Drugs that have multiple targets might be a promising combination therapy to modulate TME, especially those that could elicit both innate and adaptive immune responses against cancers.

Traditional Chinese medicine (TCM) has a long history in treating various diseases and ailments. Formula, a prescription that contains multiple drugs with different but related modes of action, is a representative of combination therapy in the system of TCM [15]. With the guidance of this philosophy, the Qin Huang formula (QHF), was grouped and used as adjuvant therapy for treating lymphoma in our hospital (Patent No.:2015103646392). Containing Scutellaria baicalensis Georgi. (Lamiaceae) (SR), Astragalus membranaceus (Fisch) Bunge (AM), *Prunella vulgaris* L. (Lamiaceae) (PV), and *Curcuma Longa* L. (Zingiberaceae) (CL), the 4-herb formula conforms to the theology of TCM in treating malignancies. Huang et al. [16] found that baicalin, an ingredient of SR, could down-regulate PI3K/Akt signaling pathway and induce apoptosis in the Burkitt lymphoma cell line. Kumagai et al. [17] demonstrated that SR and its major components, baicalein, and wogonin, could exert anti-proliferation and induce apoptosis mediated by mitochondrial damage in lymphocytic leukemia, Burkitt lymphoma, and myeloma cell lines. Studies [18–20] also showed that PV and CL have a strong antiproliferation effect upon Raji, a Burkitt lymphoma cell line, and other solid tumors. Meanwhile, it is reported that AM and its extract could protect human epithelial and endothelial cells from LPS-induced apoptosis [21] and exert a cardioprotective effect against Adriamycin-induced cardiotoxicity [22]. Thus, as a mixture of the four herbs, QHF was expected to exert an antitumor effect in B cell lymphoma as well. And our previous work [23] found that QHF (also referred to as Qin Huang mixture) has a modulating effect on the systemic immune function of lymphoma patients who have completed R-CHOP chemotherapy. Besides, we also found that when combined with R-CHOP, QHF can significantly lower the overall side effect frequencies and improve the short-term effects of R-CHOP in treating initial onset DLBCL patients [24]. In addition, we explored the effects of several major flavonoids of QHF in vitro and found that wogonin, luteolin, kaempferol, quercetin, and silymarin can inhibit the proliferation and induce apoptosis of the Raji, SU-DHL-4, and A20 cell lines (data not published), which is consistent with other studies [25–28]. Therefore, we hypothesized that QHF can enhance the antitumor effects of chemotherapy by modulating immune functions, especially the tumor immune microenvironment (TIME), of B cell NHL. In this study, a systemic pharmacological analysis was carried out to predict the targets and mechanisms of QHF in treating B cell lymphoma. Then, the antitumor effect of QHF in combination with Adriamycin (ADM) was evaluated on a murine model induced by the mouse B cell lymphoma cell line A20. Moreover, the TME of the mouse B cell lymphoma was further evaluated via IHC staining, ELISA, as well as western blot to confirm our hypothesis. Our extensive study provides experimental evidence regarding the antitumor effect of QHF + ADM combination therapy and revealed its underlying mechanisms.

## Methods

### Screening the chemical constituents of QHF and their targets

The ingredients information of QHF was searched through four databases: TCMSP [29] (https://www.tcmspw.com/tcmsp.php), DrugBank [30] (https://go.drugbank.com/#), BATMAN-TCM [31] (http://bionet.ncpsb.org.cn/batman-tcm/), and ETCM [32]^30^ (http://www.tcmip.cn/ETCM/). These databases contain information on chemical constituents and their targets of herbs. The screening criteria were set as oral bioavailability (OB) ≥30% and drug-likeness (DL) > 0.18 to collect the potential ingredients and their targets of SR, AM, PV, and CL.

B cell lymphoma-associated targets were collected from the following databases: Genecards [33] (https://www.genecards.org/), GEO [34] (https://www.ncbi.nlm.nih.gov/geo/), DisGeNET [35] (https://www.disgenet.org/), and OMIM [36] (https://www.omim.org/). As of GEO analysis, the GSE12453 dataset was utilized to draw the differentially expressed genes of DLBCL compared with naive B cells. The results with P-adjusted value < 0.05 and | log2(fold change) | > 1 were kept for further analysis. Then R platform ggplot2 [37] package was used to draw a volcano plot. Finally, with the help of the R platform VennDiagram [38] package, the common targets of QHF and B cell lymphoma were analyzed.

### The protein-protein interaction (PPI) network and drug-disease-target network building

The common targets were imported to the STRING [39] database (version 11.0, https://string-db.org) to construct the PPI network. The gene expression was correlated with others by minimum required interaction score thresholds of 0.9. Disconnected nodes in the network were hidden. The nodes in the PPI network represent the protein targets, while the edges stand for the interaction between two targets. Then, topological parameters of nodes betweenness, closeness, degree, network centrality, as well as local clustering coefficient were analyzed via R in the network. Targets with node degrees over twice the average were kept and ranked by node degree. Then the top 30 targets were counted to be the core targets. Circlize [40] package (version 0.4.13) of the R platform [41] was used to construct an herb-target chord plot to display the relationship between the herbs and the core targets. And a drug-disease-target network was constructed with the utilization of Cytoscape 3.6.0 software [42] to show the interactions of the compounds and the core targets.

### Gene ontology (GO) functional and Kyoto encyclopedia of genes and genomes (KEGG) pathway enrichment

GO and KEGG enrichment analysis was employed to further clarify the biological interpretations and signaling pathways of the 30 core targets we obtained in Part 2.2. ClusterProfiler [43], an R (version 4.1.0) package for GO and KEGG enrichment analysis tool, was utilized to access the databases of GO and KEGG. Pathview [44] package of R platform was applied to visualize KEGG pathways. The genes with adjusted *P*-value < 0.05 were retained.

### The molecular docking processing

Although the relationship between the targets and compounds was verified, the intensity of the interaction remained unclear. The computer-assistant molecular docking technology was used in this study to further investigate the ligation intensity. First, the SDF files of the compound structures were downloaded from the PubChem database (https://pubchem.ncbi.nlm.nih.gov/). Second, the protein data bank (PDB) (http://www.rcsb.org/) database was searched to obtain the molecular structures of the target proteins. The ligand-receptor compound structures were priorly chosen and modified by removing the original ligands. The pocket of the combination was constructed in the slot. Third, after adding polar hydrogen atoms, the AutoDock Vina software [45] (ver. 1.1.2, the Scripps Research Institute, U.S.) was used to achieve the binding free energy of ligands and receptors. Finally, the bonds within the ligand-receptor complexes were visualized by Ligplot software [46] (ver. 4.5.3, EBI, UK).

### Materials and reagents

The herbal formula QHF is constituted of four medicinal herbs: SR (20 g), AM (20 g), PV (15 g), and CL (10 g) at a rate of 4:4:3:2 (w/w/w/w). The herbs were obtained from Shanghai Wanshicheng Pharmaceutical Co. Ltd. (Lot Number: SR 20210406-1; AM 20210515-1; PV 20210604-1; CL 20210526-1), and the frozen powder of QHF was prepared by Shanghai Traditional Chinese Medicine Technology Co. Ltd. To obtain the extract of QHF, the herbs were first soaked in water at tenfold volume (v/w) overnight, then extract the decoction twice for 1 h each time with a tenfold volume of water to herbs (v/w). After filtration, the solution was evaporated under reduced pressure at 60 °C to a relative density of 1.15, then the extract was frozen desiccated to powder, and stored at − 20 °C for further use.

According to the pre-study of the effective dosage of QHF we conducted on mice, we found QHF (6000 mg/kg) is more effective compared with QHF (300 mg/kg) and QHF (3300 mg/kg), but tolerable as well (see Additional file 1). Therefore, the regimen of the combination group (QHF (6000 mg/kg) + ADM (5 mg/kg)) was used in this study.

### Animals and treatments

Nineteen male Balb/c mice aged 6-8 weeks were purchased from the Animals Care Centre at Shanghai Jiao Tong University. The animals were given free access to food and water and acclimatized for 1 week under standard conditions and 12 h light/dark cycle. All experimental procedures were conducted under the requirements of the research ethics Committee at Shanghai Jiao Tong University (Ethical reference no. 10687).

The mouse B cell lymphoma A20 cell line was kindly provided by the Institute of Hematology, Shanghai Ruijin Hospital, and cultured in RPMI 1640 medium (Gibco, Gaithersburg, MD, USA) containing 10% fetal bovine serum (FBS), 100 U/mL penicillin, and 100 μg/ mL streptomycin. The cells were incubated at 37 °C with 5% CO_2_. Then the cells (~ 1.0 × 10^7^ cells/mouse) were transplanted subcutaneously into the right axillary region of the mice. After 1 week or so, when the tumors reached a size of 50 mm^3^, the mice were randomly allocated into three groups as follows:

- Control group (Control): intravenously injected with 0.1 mL PBS three times on day 1, day 4, and day 8, respectively.
- Adriamycin group (ADM): received ADM (5 mg/kg) intravenously (i.v.) three times on day 1, day 4, and day 8, respectively [47].
- Combination therapy group (QHF + ADM): received 6000 mg/kg QHF dissolved in ddH_2_O intragastrically, 0.1 mL per day; ADM was administered same as ADM group.

The tumor diameters were measured routinely with a caliper. Tumor volume was estimated as follows: length × width^2^× 0.5. Besides, the tumor growth inhibition rate (TGI, %) was calculated to evaluate the anticancer effect of the two regimens.

$$\mathrm{TGI}\left(\%\right)=\left(1-\frac{tumor\ volume\ of\ treated\ group}{tumor\ volume\ of\ control\ group}\right)\ast 100\%$$

QHF was administered intragastrically (i.g.) every day throughout the experiment. On day 12, the mice were sacrificed under euthanasia with pentobarbital sodium. After dissection, the tumor of each mouse was divided into two parts, one part was kept in 4% paraformaldehyde and the other was freshly frozen in liquid nitrogen and then stored at − 80 °C.

### Immunohistochemistry (IHC) on formalin-fixed paraffin-embedded samples

Tumors were embedded in paraffin blocks and cut into tissue sections 4 μm thick. Then hematoxylin & eosin staining was processed to confirm the presence of the tumors. Besides, paraffin sections were dewaxed with xylene and rehydrated with alcohol at graded concentrations for immunohistochemical staining. Endogenous peroxidase was inactivated with 3% H_2_O_2_ for 15 minutes. Then, the slides were blocked with 5% goat serum for 20 min at 37 °C, followed by incubated with anti-CD4 (1:3000, Servicebio, China), anti-CD8 (1:1000, Servicebio, China), anti-CD206 (1:500, Servicebio, China) and anti-iNOS (SP126, 1:100, Abcam, UK) antibodies overnight at 4 °C. The next day, samples were incubated with a secondary antibody (Servicebio, China) for 1 hour at room temperature. After that, staining the samples with the ready-to-use reagent DAB kit. Finally, the sections were observed with a microscope.

The images of the tissue sections were obtained by the tissue slice digital scanner. The Servicebio image analysis system was used to automatically analyze and calculate the number of weak, medium, and strong positive cells in the measurement area (negative for no coloring, score 0 points; weak positive for light yellow, score 1 point; medium positive for brownish yellow, score 2 points; strong positive for tan, score 3 points). The positive cell rate indicator was used to assess the percentage of positive cells [48].$\mathrm{positive}\ \mathrm{cell}\ \mathrm{rate}\ \left(\%\right)=\frac{number\ of\ positive\ cells}{\ number\ of\ total\ cells}\ast 100\%.$

### Enzyme-linked immunosorbent assay (ELISA) on FFPE samples

The total protein was extracted from the freshly frozen tumor tissues previously stored. First, 1 ml of RIPA buffer was prepared for every 0.1 g of tissue and protease and phosphatase inhibitors were added to a final 1x concentration. The tissues were ground under 60 Hz for 4 cycles. Then, the homogenate was centrifuged at 12500 rpm at 4 °C for 15 minutes. After this, the supernatant was transferred to a clean tube for ELISA. According to the manufacturer’s instructions, commercially available ELISA kits (MultiSciences (LIANKE) Biotech Co., Ltd., Hangzhou, China) were applied for mouse protein expression detection of CXCL10, IL-2, IL-17, IL-6, TGF-β, and IFN-γ levels.

### Western blotting

Three freshly frozen samples of each group were also homogenized in RIPA buffer supplemented with proteinase/phosphatase inhibitors. After centrifuging, the protein concentration was determined with a BCA protein assay kit (Beyotime, Shanghai, China). Twenty μg protein from each sample was subjected to 12% SDS-PAGE and electrotransferred to 0.22uM PVDF membranes. Then, the membranes were subjected to blocking buffer (Beyotime, Shanghai, China), followed by incubation overnight at 4 °C with primary antibodies (1:1000, Abcam, U.S.) against p38 MAPK, p- ERK, TLR2, and β-actin. After that, the probed membranes were washed with TBST three times, followed by incubation with secondary antibodies (1:5000, Abcam, U.S) for 1 h at room temperature. The protein signals were detected using a chemiluminescence ECL assay kit and imaged using a Tanon 5200 imaging system. β-actin was served as the internal reference. The expression levels of proteins were calculated by Image J platform.

### ^18^F-FDG PET-CT scan

One mouse of each group was picked out to receive the ^18^F-FDG PET-CT scanning on the first day (d1) and the last day (d8) of therapy, respectively. The combination group underwent another ^18^F-FDG PET-CT scan on the 7th day after discontinuing therapy. PET/CT imaging was performed on the Inveon MM Platform (Siemens Preclinical Solutions, Knoxville, Tennessee, USA), which was equipped with a computer-controlled bed and 8.5 cm transaxial and 5.7 cm axial fields of view (FOV). After fasting overnight for 8 h and being anesthetized under pentobarbital sodium, three mice received an intravenous infusion of 100-200 uCi ^18^F-FDG. The scanning process was performed with Inveon Acquisition Workplace (IAW) 1.5.0.28. Before the PET scan, the CT X-ray for attenuation correction was scanned for 10 minutes with a power of 80 Kv and 500 uA and an exposure time of 1100 ms. Then, 10-minute static PET scans were acquired, followed by images reconstruction with an OSEM3D (Three-Dimensional Ordered Subsets Expectation Maximum) algorithm. The 3D regions of interest (ROIs) were drawn over the right axillary region guided by CT, with the tracer uptake measured by the Inveon Research Workplace (IRW) 3.0. Individual quantification of the ^18^F-FDG uptake, as well as the max uptake values (SUV_max_) of the mice, were determined.

### Statistical analysis

The obtained data were expressed as mean ± standard errors. Statistical analysis was performed on the platform of GraphPad Prism 9 (GraphPad Software, San Diego, CA, USA). Comparisons among multiple groups were performed by one-way ANOVA with Tukey’s multiple comparisons test. Comparisons between two groups were assessed using Student’s unpaired t-tests. A *p*-value < 0.05 was considered significant.

## Results

### Active components and hub genes of QHF in treating B cell lymphoma

**Fig. 1:**
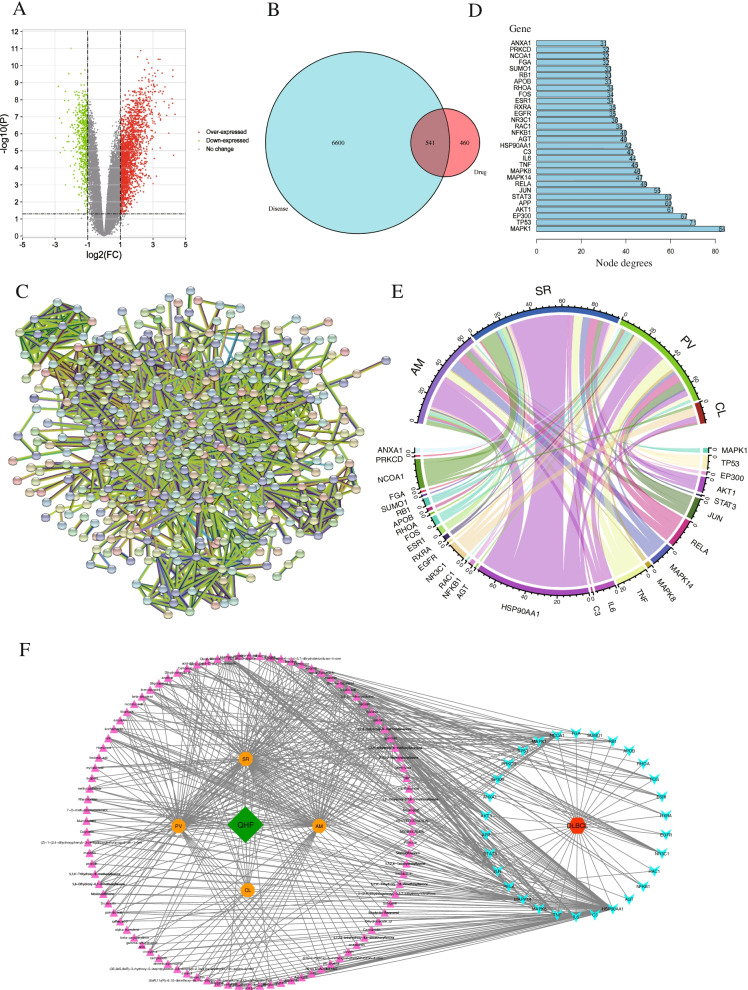
Active ingredients and core targets screening. **A**. The volcano plot displayed the significance and the fold change of differentially expressed genes acquired from the GEO database; The red ones represented up-regulated DEGs (log2(fold change) > 1), while the green ones represented down-regulated DEGs (log2(fold change) < − 1). **B**. The Venn diagram demonstrates the overlapping of network pharmacology-based targets of QHF components (red color) and transcriptionally dysregulated genes of B-cell lymphoma (turquoise color). **C**. The Protein-Protein-Interaction (PPI) network of the core genes. **D**. The core targets and their node degrees. **E**. The herb-target chord chart displays the relationship of the most involved targets and herbs. **F**. The drug-disease-target network indicated the relationship between the drugs, compounds, and targets. The pink triangles of the left peripheral circle represented the compounds of QHF, while the four orange octagons represented the four herbs of QHF. The blue arrows of the right peripheral circle represented the core targets of B cell lymphoma

A total of 271 active ingredients of QHF with OB ≥ 30% and DL > 0.18 were obtained. In the GEO database, the samples of GSM312858-312869 (DLBCL), GSM312870-312876 (naive B-cells), and GSM312877-312886 (memory B cells) from GSE12453 were selected. The volcano plot shows the relationship between the significance and the foldchange of differentially expressed genes (DEGs). They were highlighted by the red and green dots (Fig. 1A). Filtered by p-adjusted value < 0.05, a total of 7141 transcriptionally dysregulated genes of B-cell lymphoma were discovered. The Venn diagram shows the overlapping of QHF (red color) and DLBCL (turquoise color) related targets (Fig. 1B). Finally, we obtained a total of 541 common targets. To explore the interactions between the targets of QHF, a PPI network was constructed with the STRING database. After importing 541 common targets into the database, a total of 539 nodes and 2807 edges were built with an average node degree of 10.4 (Fig. 1C). By topology analysis of the interactions in the PPI network, we obtain a bar plot displaying the core targets and their node degrees (Fig. 1D). According to this figure, MAPK1/8/14, TP53, EP300, AKT1, APP, STAT3, JUN, RELA, TNF, IL6, C3, HSP90AA1, AGT, NFKB1, RAC1, NR3C1, EGFR, RXRA, ESR1, FOS, RHOA, APOB, RB1, SUMO1, FGA, NCOA1, PRKCD, and ANXA1 were the top 30 targets with the most nodes degree. Among these core targets, MAPK1/ERK2, MAPK8/JNK1, and MAPK14/p38 consist of the MAPK signaling pathway, which regulates diverse biological functions, including cell growth, differentiation, and survival. JUN is one of the components of AP-1, which can be phosphorylated by MAPK8/JNK1 and induce the expression of inflammatory cytokines, including IL-6 and TNF-α. NFKB and AKT1 are the key molecules in the NF-κB signaling pathway and PI3K-Akt signaling pathway, respectively. The above three pathways are the downstream pathway of TLRs.

To further investigate the relationship between herbs, components, and hub targets, the R platform was utilized to form a chord chart (Fig. 1E). According to the plot, there were 64, 96, 72, and 13 edges in AM, SR, PV, and CL, respectively. The herb which related to most targets was SR (degree = 96). In addition, the relationship between drugs and the disease was described by the drug-disease-target network (Fig. 1F). In this network, there were 220 nodes and 625 edges. Quercetin in AM and PV (degree = 20), kaempferol in AM and PV (degree = 12), luteolin in PV (degree = 9), and wogonin in SR (degree = 9) ranked the top four compounds that connected most quantity of targets of DLBCL. In CL, stigmasterol relates to the most targets in the network. Among these five active components, four belong to flavonoids and only stigmasterol of CL belongs to sterol. The structural formula and other important parameters of the major active components of QHF were shown in Table 1.

**Table 1:**
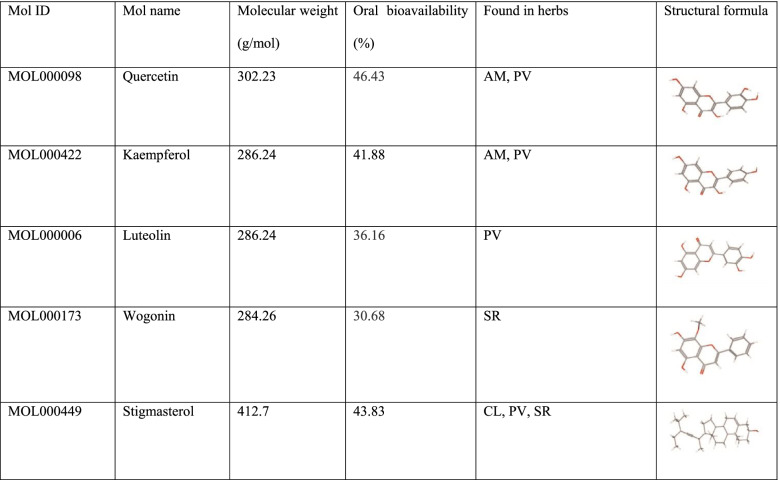
The structural formula and other important parameters of the major active components of QHF.

^*^Note: SR for Scutellaria baicalensis Georgi. (Lamiaceae); AM for Astragalus membranaceus (Fisch) Bunge; PV for *Prunella vulgaris* L. (Lamiaceae); CL for *Curcuma Longa* L. (Zingiberaceae).

### Predicted mechanisms of QHF in treating B cell lymphoma

**Fig. 2:**
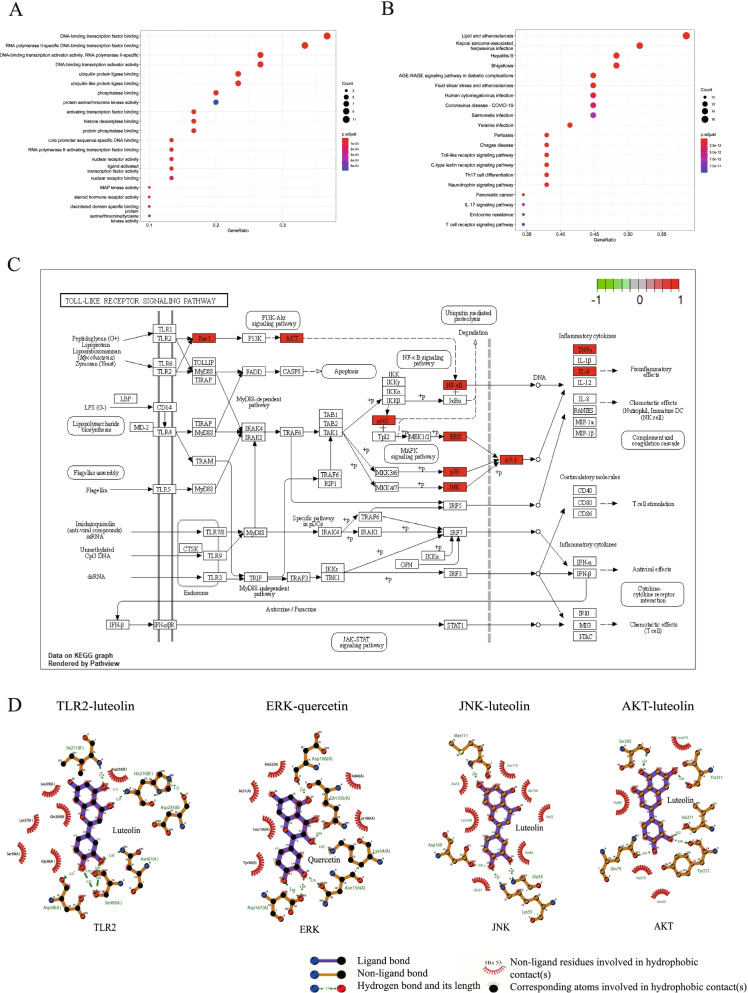
Predicted mechanisms of QHF in treating B cell lymphoma. **A**. The dot plot of GO functional enrichment of QHF. **B**. The dot plot of KEGG pathway enrichment analysis of QHF. **C**. The KEGG pathway diagram of Toll-like receptor signaling pathway [49–51]. It shows that QHF could target AKT, NF-κB, p38, ERK, and JNK to activate PI3K/Akt signaling pathway, MAPK, and NF-κB signaling pathways. **D**. Representative images of molecular docking show that the active components of QHF bind to TLR2, ERK, JNK, and AKT primarily with hydrogen bonds

As mentioned above, the top 30 core targets (see Additional file 2) were input for GO enrichment and KEGG pathway enrichment analyses to show the biological activity and potential mechanisms of the hub genes. The most enriched functions in the GO analysis are shown in Fig. 2A. In detail, the top three terms in the GO biological processes were DNA-binding transcription factor binding (GO:0140297), RNA polymerase II-specific DNA-binding transcription factor binding (GO:0061629), DNA-binding transcription activator activity, RNA polymerase II-specific (GO:0033613).

KEGG pathways enrichment analysis was conducted to further explore the possible functions of the targets. In this section (see Additional file 3), Lipid and atherosclerosis (hsa05417), AGE-RAGE signaling pathway in diabetic (hsa04933), Kaposi sarcoma-associated herpesvirus infection (hsa05167), Hepatitis B (hsa05161), Fluid shear stress and atherosclerosis (hsa05418), Pertussis (hsa05133), Yersinia infection (hsa05135), Shigellosis (hsa05131), Chagas disease (hsa05142), Toll-like receptor signaling pathway (hsa04620), C-type lectin receptor signaling pathway (hsa04625), Th17 cell differentiation (hsa04659), Pancreatic cancer (hsa05212), Neurotrophin signaling pathway (hsa04722), Human cytomegalovirus infection (hsa05163), Coronavirus disease-COVID-19 (hsa05171), IL-17 signaling pathway (hsa04657), Endocrine resistance (hsa01522), Salmonella infection (hsa05132), T cell receptor signaling pathway (hsa04660), TNF signaling pathway (hsa04668) were significantly enriched (Fig. 2B). The KEGG pathway diagram disclosed that a series of target genes constituted the Toll-like receptor signaling pathway, including AKT, NFKB, ERK, JNK, IL6, and p38 MAPK (Fig. 2C). Therefore, it is reckoned that QHF exerts therapeutic effects through regulating immune-related functions.

Moreover, the molecular docking verified a strong affinity of active components binding to targets in the predicted pathway (Fig. 2D). The docking parameters of each target and binding free energies were listed in Table 2. The results indicated that flavonoids of QHF, including quercetin, luteolin, kaempferol, and wogonin, had a strong affinity with core targets in terms of binding free energy. Interestingly, ASTX029, the novel inhibitor of ERK [52], binds to ERK with a binding free energy of − 8.3, identical to that of quercetin, suggesting a high affinity of quercetin binding with ERK. The ligands and receptors were bound mainly by hydrogen bonds, which is positively related to the binding affinity. For example, luteolin could bind to TLR2, AKT, and JNK closely, with 6, 4, and 5 hydrogen bonds, respectively, and quercetin bound to ERK with 6 hydrogen bonds. Apart from the hydrogen bonds, the secondary effects were hydrophobic interaction of amino. These results further revealed the affinity of the active components of QHF binding to the transcriptionally dysregulated genes of B-cell lymphoma, which is the foundation of drugs to take effect.

**Table 2:** The molecular docking parameters

Targets	PDB ID	Ligands	Size of cube (X * Y * Z) (nm^3^)	Center grid box (X, Y, Z)	Binding free energy (kcal/mol)
TLR2	2Z80	Luteolin	100*100*100	−1.516	−8.3
				−14.704	
				−14.946	
ERK	7AUV	Quercetin	96*80*124	0.759	−8.3
				4.044	
				37.087	
AKT	3O96	Luteolin	104*90*126	52.684	−9.9
				76.365	
				34.530	
JNK	2NO3	Luteolin	104*90*126	15.902	−8.8
				58.495	
				0.023	

### Antitumor effects of QHF in combination with ADM in vivo

**Fig. 3:**
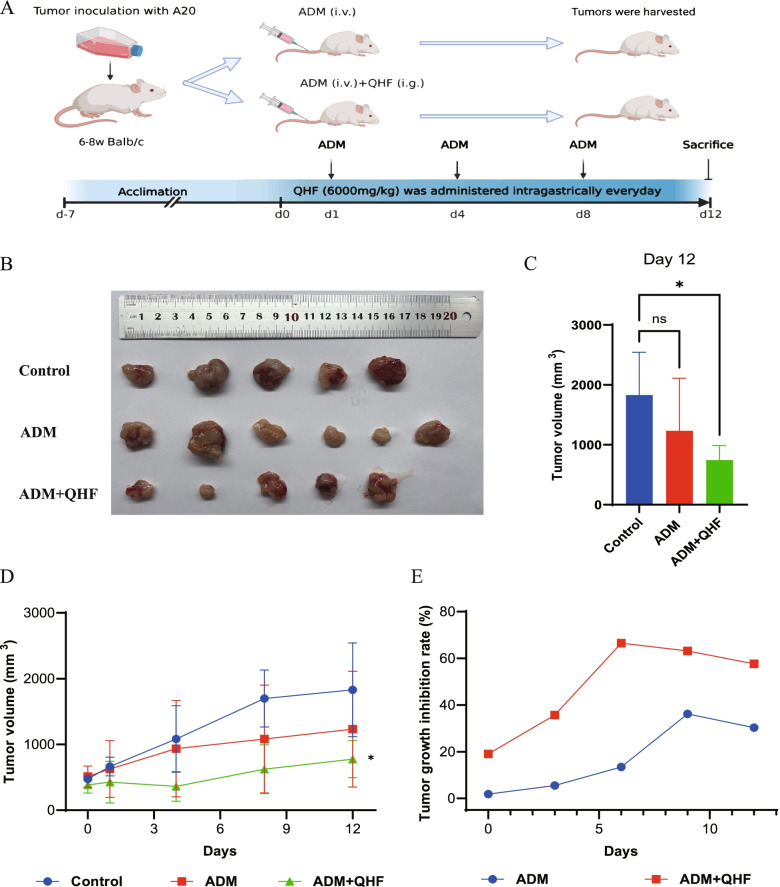
QHF + ADM significantly inhibits the growth of A20 in the tumor-bearing mice model. **A**. Scheme of the animal experiment protocol. **B**. Photographs of all the tumor blocks collected from the Control group (*n =* 5), ADM group (*n =* 6), and QHF + ADM group (*n =* 5). **C**. Tumor volume of the tumor blocks collected from mice on day 12. It showed that QHF + ADM group (*n =* 5) significantly lowered tumor volume when compared with the control group (*n =* 5, *p* < 0.05). But there is no significance between the ADM group (*n =* 6, *p* > 0.05) and the control group (*n =* 5, p > 0.05). **D**. The tumor volume changes with time of the three groups. The tumor growth of the QHF + ADM group (*n =* 5) was the slowest of the three groups, followed by the ADM group (*n =* 5). **E**. Tumor growth inhibition rate (TGI, %) of the treatment groups. It showed that QHF + ADM (*n =* 5) could significantly inhibit tumor growth in an A20 murine model (vs. ADM (*n =* 5), *p <* 0.01). Data are presented as the mean ± standard errors, *p < 0.05

In this study, we first investigated the in vivo effects of QHF and Adriamycin (ADM) combination therapy on the growth of B cell lymphoma in A20 tumor-bearing mice. The study scheme is depicted in Fig. 3A. Mice bearing subcutaneous A20 tumors in QHF + ADM (*n =* 5) and ADM (*n =* 6) groups were compared with mice in the control group (*n =* 5). The tumor volume on day 12 was 744.07 mm^3^ (QHF + ADM group), 1275.23 mm^3^(ADM group), and 1829.89 mm^3^ (control group), respectively (Fig. 3B). Compared with the control group, the tumor volume of the QHF + ADM group was significantly lowered (vs. control group, *p <* 0.05). However, the ADM group showed no significant difference (vs. control group, *p >* 0.05), but exhibited a declining trend (Fig. 3C, D). There was a significant difference in TGI between QHF + ADM group and ADM group on day 12 (*p <* 0.01) (Fig. 3E).

### QHF in combination with ADM increases tumor-infiltrating CD4+ and CD8+ T cells

**Fig. 4:**
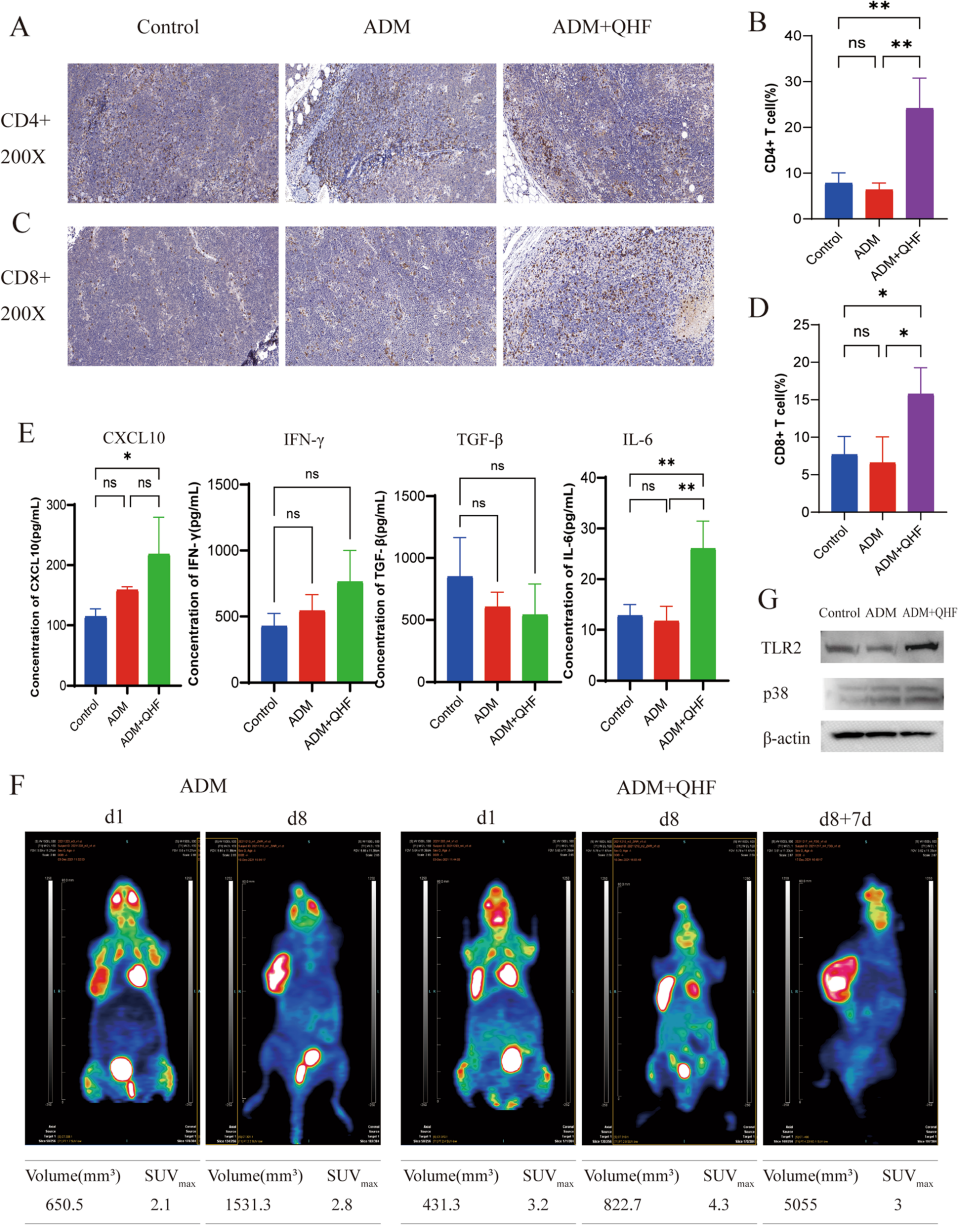
QHF + ADM recruited CD4+ and CD8+ T cells in the A20 tumor sites. **A**. Representative images of IHC staining for CD4+ T cell among the three groups (200×). **B**. Bar plot of the positive cell rate of CD4+ T cells of the control group (*n =* 3), ADM group (*n =* 3), and QHF + ADM group (*n =* 3). **C**. Representative images of IHC staining for CD8+ T cells among the three groups (200×). **D**. Bar plot of the positive cell rate of CD8+ T cells of the control group (*n =* 3), ADM group (*n =* 3), and QHF + ADM group (*n =* 3). **E**. Bar plot of TGF-β, IFN-γ, CXCL10, and IL-6 levels based on ELISA of the control group (*n =* 3), ADM group (*n =* 3), and QHF + ADM group (*n =* 3).**F**. ^18^F FDG PET-CT scan of the ADM group (*n =* 1), and QHF + ADM group (*n =* 1). **G**. Expression of TLR2, p38 MAPK, and β-actin by western blot. Data are presented as the mean ± standard errors, *p < 0.05; **p < 0.01

In light of the systematic pharmacology research we did previously, we predicted that QHF probably exerts such tumor inhibition effect through modulating immune functions by activating the Toll-like receptor (TLR) signaling pathway. To verify the prediction, the tumors harvested were used to explore the differences in TME among the three groups. Sixteen tumor-bearing mice (three groups) were used, and their tumor blocks were stained for tumor-associate macrophages (TAMs) and tumor-infiltrating lymphocytes (TILs) via IHC. Chemokines and cytokines, including CXCL10, IL-2, IL-17, IL-6, IFN-γ, and TGF-β were also tested with ELISA. The IHC staining showed that there is no significance among the three groups in the proportion of M1 macrophages and M2 macrophages, but the combination group displayed a tendency to decline in the proportion of M2 macrophages (see Additional file 4). Interestingly, the proportions of CD4+ and CD8+ T-cells in the tumor tissues were significantly higher in the combination therapy group than in the ADM monotherapy group (Fig. 4A-D). As for the CD4+ T-cell ratio, the QHF + ADM group showed a significant difference when compared with the control group (*p <* 0.01) and the ADM monotherapy group (*p <* 0.01) (Fig. 4A, B). Besides, there was a significant increase of CD8+ T-cells in the combination group (vs. control group, *p <* 0.05; vs. ADM group, *p <* 0.05) (Fig. 4C, D). The ELISA revealed that mice in the combination group with QHF + ADM had higher levels of CXCL10 (v.s. Control, *p <* 0.05) and IL-6 (v.s. Control, *p <* 0.01). Meanwhile, the levels of TGF-β displayed a declining tendency, although the difference was not significant (Fig. 4E). Neither significant changes nor tendencies were observed for IL-2 and IL-17 (data not shown, see Additional file 5). The PET-CT images showed that compared with the pretreatment scans, the posttreatment mice had a relatively higher SUV_max_ value (Fig. 4F). As for the mouse receiving ADM+ QHF, seven days after the therapy was discontinued, the volume of tumor enormously increased but the SUV_max_ value lowered from 4.3 to 3, instead. These results suggested that combination therapy could remarkably enhance the level of tumor-infiltrating lymphocytes, especially the CD4+ and CD8+ T cells.

### QHF exerts an antitumor effect by targeting the toll-like receptor signaling pathway

The protein levels of TLR2, p38 MAPK, and β-actin expression were detected by western blot in the present study. As shown in Fig. 4G, compared with the Control group (*n =* 3) and the ADM group (*n =* 3), the combination group (*n =* 3) significantly increased the expressions of TLR2 (v.s. Control group, *p* < 0.001; v.s. ADM group, *p* < 0.0001) and p38 MAPK (v.s. Control group, p < 0.001) (see Additional file 6).

## Discussion

B cell non-Hodgkin lymphoma is a highly heterogeneous neoplasm, and its most common subtype is diffuse large B cell lymphoma. Although it is curable to most patients, there are still 30-40% recurrence or refractory cases [53]. And cancer immunotherapies are initially used in these settings. The TME, especially the immune landscape within it, is found to be crucial in the responses to the immunotherapies [54]. The TME of B-cell lymphoma mainly consists of T cells, macrophages, natural killer (NK) cells, stromal cells, blood vessels, and extracellular matrix [55, 56]. A combination therapy targeting TME has proven efficacy and displayed great potential for novel immunotherapies. As a traditional formula, QHF consists of four different herbs with various functions. In this study, we verified the tumoricidal effect of QHF in combination with ADM using a murine model and explored its underlying mechanisms with the guidance of systemic pharmacology.

Systemic pharmacology is a comprehensive approach to clarify the active compounds and drug targets, as well as to predict the mechanisms of traditional Chinese medicinal formulas. Our work displayed that QHF has 541 significant targets in B cell lymphoma, among which MAPK1/ERK2 and AKT1 are mostly enriched. Based on KEGG pathway enrichment analysis, we discovered that the TLR signaling pathway, which plays a critical role in activating both innate and adaptive immune responses, was one of the major pathways that QHF might trigger. In addition, we filtered out five active components of QHF, i.e., quercetin, luteolin, kaempferol, wogonin, and stigmasterol, that might be the most promising ingredients to represent QHF. The molecular docking technique also authenticated the ligation of the five components and targets.

Our murine model built with an A20 cell line showed that compared with ADM monotherapy, the QHF + ADM group could significantly inhibit the tumor growth. Meanwhile, the tumor-infiltrating CD4+ and CD8+ T cells of the QHF + ADM group were remarkably increased. Research demonstrated that anti-CD20 mAb, such as rituximab, could achieve a long-lasting “vaccinal effect” with the presence of both CD4+ and CD8+ T cells [57, 58], indicating the potential value of QHF as an add-on therapy to the R-based therapy for lymphoma patients. In this study, one mouse from each group was picked out to receive the ^18^F-FDG PET-CT scan before and after the treatment, respectively. Notably, although the posttreatment tumor volume of the combination group is smaller than the ADM group, the SUV_max_ of the combination group is higher. What’s more, after the treatment was discontinued for 1 week, the SUV_max_ of the combination group decreased to a level lower than that of pretreatment. Evidence showed that SUV_max_ is positively related to the TILs and TAMs [59, 60]. And a provoking study published in *Nature* this year has proven that it is the myeloid cells, followed by T cells, that uptake most FDG rather than tumor cells [61]. Therefore, the dynamic changes of SUV_max_ of the combination group might indicate the level changes of infiltrating T cells. The ^18^F-FDG PET-CT scan provided an additional description of the increased infiltrating T cells in the QHF + ADM group.

According to the immunophenotype of TME, tumors can be divided into “hot” tumors and “cold” tumors. Hot tumors present with rich TILs, whose initial immune response is obstructed by upregulation of immune checkpoints or immunosuppressive cells. Anti-PD-1/PD-L1 antibodies block tumor immune evasion by protecting CD8+ T cells from PD-1 mediated cell death [62, 63]. However, such ICI has limited efficacy in ‘cold’ tumors, where immune cells are less abundant or completely absent in TME [64]. Mullins et al. [65] found that TLR agonists tuned TME to inflamed immunophenotype by activating CD8+ T cells, thus enhancing the antitumor effect of ICIs. This has highlighted the promising value of TLR agonists. TLR is a big family consisting of TLR2, TLR4, TLR7, TLR8, and other receptors. TLR signaling pathway starts with TLR receptor and ligand recognition, followed by TLR dimerization and a signaling cascade to produce inflammatory molecules. In this study, we first predicted that the active components of QHF bind to TLR2 with hydrogen bonds via molecular docking methods. In theory, when the components of QHF bind to TLR2 and activate the TLR2 signaling pathway, the constitutive MAPK or NF-κB signaling pathway will be triggered to express various cytokines like IL-6, TNF-α, and IFNs. It is also reported that activation of TLR2 can reverse mouse Treg suppression, promote and generate efficient memory T cells by increasing IFN-γ level, and enhance the cytotoxic activity of CD8+ T cells [66]. Besides, ADM can activate p38 to regulate the expression of topoisomerase II, inducing apoptosis of lymphoma cells. Therefore, QHF + ADM could enhance the antitumor effect via different mechanisms theoretically. In the present study, the IL-6 level was up-regulated in the QHF + ADM group, but the levels of TNF-α and IFN-γ were not remarkably changed. As the IL-6 production regulation is controlled through both transcriptional and posttranscriptional mechanisms [67], it remains elusive whether the increased IL-6 is the result of TLR signaling pathway activation or not. But the increase in CD4+ and CD8+ T cells in TME, as well as up-regulated CXCL10 and TLR2 in the combination group, indicated QHF + ADM could modulate TME by targeting TLR2.

As aforementioned, the immune cells within TME can interact with the tumor cells directly or through chemokines and cytokines to impact the behavior of the tumor and response rate [68]. A variety of chemokines are expressed by immune cells, including CCR7, CXCL9, CXCL10, CCL2, and IFN-γ. Secreted by effector T cells, IFN-γ can induce the expression of CXCL10, which in return attracts even more T cells into the TME, thus forming a positive feedback loop [69–73]. Despite IFN-γ, TLR agonists also elicit cytokines and chemokines expression in multiple cells [65]. In a melanoma model, TLR2/6 agonists + IFN-γ can induce CXCL10 production, leading to more T cells migrating into TME [74]. In our study, the combination group displayed upregulated levels of TLR2 and CXCL10, indicating that QHF might target TLR signaling pathway to promote CXCL10 expression with the presence of IFN-γ, and consequently increased T cell infiltration and IFN-γ expression in return. Transforming growth receptor beta (TGF-β), expressed by both tumor cells and tumor-associated cells, directly inhibits T cell proliferation and activation while promoting the survival of immunosuppressive Tregs. Therefore, in the context of TME, upregulated TGF-β may lead to T cell dysfunction and limited efficacy of cancer immunotherapies [75]. Moreover, with the presence of IL-6, TGF-β may also promote CD4+ T cell differentiation along a Th17 pathway, which was predicted in the KEGG enrichment analysis as mentioned before. Although IL-6 is associated with lymphoma proliferation and poor prognosis, it is reported that IL-6 can prevent apoptosis of T cells by upregulation of Bcl-2 and promote T cell proliferation via the TCR pathway [76]. Also, evidence shows that increased IL-6 is associated with highly functional CAR T cells and their expansion [77]. Consistent with these findings, we observed a downregulated level of TGF-β, as well as an upregulated level of IL-6 in the combination group, suggesting that QHF may aid to restore T cell function and survival in the TME. Taken together, we speculated that QHF took effect through modulating the TIME via targeting the TLR signaling pathway.

## Conclusion

QHF in combination with ADM improves the anti-lymphoma effect of ADM by modulating TME. Besides, with a method of systematic pharmacology, the active components of QHF were identified. This research established a scientific method for analyzing chemical components and verifying predicted mechanisms of Chinese herbal medicine.

## Supplementary Information

**Additional file 1: Figure S1.** Pre-study data of the effective dosage of QHF. A.The image of the tumor blocks of the three groups. B.Thebodyweight of the mice in three groups during the experiment. C. The tumor blocks weight of the three groups. Compared with the LD group (*n=*5), the tumor weight was significantly decreased in the HD group (*n=*5, **p<0.01). Compared with the MD group (*n=*5), the tumor weight was remarkably lowered in the HD group (*n=*5, *p<0.05)

**Additional file 2: Table S1.** Total input gene list

**Additional file 3: Table S2.** KEGG pathway enrichment results

**Additional file 4: Figure S2.** IHC stainingsfor M1 and M2 macrophages. A. Representative images of IHC staining for M1 macrophages among the three groups (200×). B. Bar plot of the positive cell rate of M1 macrophages of the control group (*n=*3), ADM group (*n=*3), and QHF+ADM group (*n=*3). C. Representative images of IHC staining for M2 macrophages among the three groups (200×). D. Bar plot of the positive cell rate of M2 macrophages of the control group (*n=*3), ADM group (*n=*3), and QHF+ADM group (*n=*3).Ns: p>0.05

**Additional file 5: Figure S3.** ELISA results of IL-2 and IL-17. A. Concentration of IL-2 of the control group (*n=*3), ADM group (*n=*3), and ADM+QHF group (*n=*3). There is no significant difference between the three groups (p>0.05). B. Concentration of IL-17 of the control group (*n=*3), ADM group (*n=*3), and ADM+QHF group (*n=*3). There is no significant difference between the three groups (p>0.05)

**Additional file 6: Figure S4.** Image J results of the WB bands. A. Bar plot of the expression of TLR2. Compared with the Control group (*n=*3) and the ADM group (*n=*3), the ADM+QHF group (*n=*3) significantly increased the expressions of TLR2 (v.s. Control group, ***p<0.001; v.s. ADM group, ****p<0.0001). B. Bar plot of the expression of p38.Compared with the Control group (*n=*3), the level of p38 MAPK was remarkably increased in ADM group (*n=*3, ***p<0.001) and ADM+QHF group (*n=*3, ***p<0.001)

## Data Availability

The main data of this study are available from the corresponding author on reasonable request. The datasets supporting the conclusions of this article are available in the public database from TCMSP, DrugBank, BATMAN-TCM, ETCM, GEO, DisGeNET, GeneCards, and OMIM.

## Abbreviations

QHF: Qin Huang formula
ADM: Adriamycin
TME: Tumor microenvironment
NHL: Non-Hodgkin lymphoma
DLBCL: Diffuse large B cell lymphoma
ICI: Immune checkpoint inhibitor
cHL: Classical Hodgkin Lymphoma
TCM: Traditional Chinese medicine
SR: Scutellaria baicalensis Georgi. (Lamiaceae)
AM: Astragalus membranaceus (Fisch) Bunge
PV: *Prunella vulgaris* L. (Lamiaceae)
CL: *Curcuma Longa* L. (Zingiberaceae)
TIME: Tumor immune microenvironment
OB: Oral bioavailability
DL: Drug-likeness
PPI: Protein-protein interaction
GO: Gene Ontology
KEGG: Kyoto Encyclopedia of Genes and Genomes
PDB: Protein data bank
FBS: Fetal bovine serum
TGI: Tumor growth inhibition rate
IHC: Immunohistochemistry
ELISA: Enzyme linked immunosorbent assay
FOV: Fields of view
IAW: Inveon Acquisition Workplace
ROI: Regions of interest
OSEM3D: Three-dimensional ordered subsets expectation maximum
MAP: Maximization/Maximum a Posteriori
IRW: Inveon Research Workplace
SUV: Standardized uptake values
DEG: Differentially expressed gene
TLR: Toll-like receptor
TAM: Tumor-associated macrophage
TIL: Tumor-infiltrating lymphocyte
NK: Natural killer
TGF-β: Transforming growth receptor beta.

## References

[1] Sung H, Ferlay J, Siegel RL, et al. Global cancer statistics 2020: GLOBOCAN estimates of incidence and mortality worldwide for 36 cancers in 185 countries. CA Cancer J Clin. Published online February 4, 2021. 10.3322/caac.21660 33538338

[2] SEER Cancer Stat Facts. Non-Hodgkin Lymphoma. Bethesda: National Cancer Institute. https://seer.cancer.gov/statfacts/html/nhl.html

[3] Coiffier B, Thieblemont C, Van Den Neste E (2010). Long-term outcome of patients in the LNH-98.5 trial, the first randomized study comparing rituximab-CHOP to standard CHOP chemotherapy in DLBCL patients: a study by the Groupe d’Etudes des Lymphomes de l’Adulte. Blood.

[4] Camicia R, Winkler HC, Hassa PO (2015). Novel drug targets for personalized precision medicine in relapsed/refractory diffuse large B-cell lymphoma: a comprehensive review. Mol Cancer.

[5] Wang L, Li LR, Young KH (2020). New agents and regimens for diffuse large B cell lymphoma. J Hematol Oncol.

[6] Anderson JK, Mehta A (2019). A review of chimeric antigen receptor T-cells in lymphoma. Expert Rev Hematol.

[7] Neelapu SS (2019). Managing the toxicities of CAR T-cell therapy. Hematol Oncol.

[8] Vari F, Arpon D, Keane C (2018). Immune evasion via PD-1/PD-L1 on NK cells and monocyte/macrophages is more prominent in Hodgkin lymphoma than DLBCL. Blood..

[9] Xu-Monette ZY, Zhou J, Young KH (2018). PD-1 expression and clinical PD-1 blockade in B-cell lymphomas. Blood.

[10] Tumeh PC, Harview CL, Yearley JH (2014). PD-1 blockade induces responses by inhibiting adaptive immune resistance. Nature..

[11] Ji RR, Chasalow SD, Wang L (2012). An immune-active tumor microenvironment favors clinical response to ipilimumab. Cancer Immunol Immunother.

[12] Ager A, Watson HA, Wehenkel SC, Mohammed RN (2016). Homing to solid cancers: a vascular checkpoint in adoptive cell therapy using CAR T-cells. Biochem Soc Trans.

[13] Lenz G, Wright G, Dave SS (2008). Stromal gene signatures in large-B-cell lymphomas. N Engl J Med.

[14] Wang L, Li LR (2020). R-CHOP resistance in diffuse large B-cell lymphoma: biological and molecular mechanisms. Chin Med J.

[15] Wang L, Zhou GB, Liu P (2008). Dissection of mechanisms of Chinese medicinal formula Realgar-indigo naturalis as an effective treatment for promyelocytic leukemia. Proc Natl Acad Sci U S A.

[16] Huang Y, Hu J, Zheng J (2012). Down-regulation of the PI3K/Akt signaling pathway and induction of apoptosis in CA46 Burkitt lymphoma cells by baicalin. J Exp Clin Cancer Res.

[17] Kumagai T, Müller CI, Desmond JC, Imai Y, Heber D, Koeffler HP (2007). Scutellaria baicalensis, a herbal medicine: anti-proliferative and apoptotic activity against acute lymphocytic leukemia, lymphoma and myeloma cell lines. Leuk Res.

[18] Yin DT, Lei M, Xu J (2017). The Chinese herb Prunella vulgaris promotes apoptosis in human well-differentiated thyroid carcinoma cells via the B-cell lymphoma-2/Bcl-2-associated X protein/caspase-3 signaling pathway. Oncol Lett.

[19] Liu X, kui, Wang L, Zhang M zhi. (2010). Involvement of JNK and caspase-3 in human lymphoma cell apoptosis induced by Prunella vulgaris. Zhonghua Yi Xue Za Zhi.

[20] Zhao Q, Guan J, Qin Y (2018). Curcumin sensitizes lymphoma cells to DNA damage agents through regulating Rad51-dependent homologous recombination. Biomed Pharmacother.

[21] Wu X, Zhou W, Wei Q, Chen P, Li Y (2018). Cytoprotective effects of the medicinal herb Astragalus membranaceus on lipopolysaccharide-exposed cells. Mol Med Rep.

[22] Abushouk AI, Ismail A, Salem AMA, Afifi AM, Abdel-Daim MM (2017). Cardioprotective mechanisms of phytochemicals against doxorubicin-induced cardiotoxicity. Biomed Pharmacother.

[23] Zhu W, Zhao X, Ruan M, Lv L, Zhao W, Shen X (2018). Effects of Qinhuang mixture on immune function of patients with diffuse large B-cell lymphoma. Chinese Traditional Patent Med.

[24] Zhu W, Zhao X, Lv L (2016). Randomized controlled clinical study in the treatment of high-risk diffuse large B-cell lymphoma with Qinhuang mixture in the patients. W J Integrated Traditional West Med.

[25] Gharbaran R, Shang E, Onwumere O, Codrington N, Sarpong ED, Redenti S (2020). Luteolin induces cytotoxicity in mix cellularity classical Hodgkin’s lymphoma via caspase activated-cell death. Anticancer Res.

[26] Soofiyani SR, Hosseini K, Forouhandeh H (2021). Quercetin as a novel therapeutic approach for lymphoma. Oxidative Med Cell Longev.

[27] Wu X, Liu P, Zhang H (2017). Wogonin as a targeted therapeutic agent for EBV (+) lymphoma cells involved in LMP1/NF-κB/miR-155/PU.1 pathway. BMC Cancer.

[28] Xu PP, Zuo HQ, Zhou RF, Chen B, Ouyang J (2018). Wogonin inhibits growth of mantle cell lymphoma cells through nuclear factor-κB signaling pathway. Chin Med J.

[29] Ru J, Li P, Wang J (2014). TCMSP: a database of systems pharmacology for drug discovery from herbal medicines. J Cheminformatics.

[30] Wishart DS, Feunang YD, Guo AC (2018). DrugBank 5.0: a major update to the DrugBank database for 2018. Nucleic Acids Res.

[31] Liu Z, Guo F, Wang Y (2016). BATMAN-TCM: a bioinformatics analysis tool for molecular mechANism of traditional Chinese medicine. Sci Rep.

[32] Xu HY, Zhang YQ, Liu ZM (2019). ETCM: an encyclopaedia of traditional Chinese medicine. Nucleic Acids Res.

[33] Stelzer G, Rosen N, Plaschkes I, et al. The GeneCards suite: from gene data mining to disease genome sequence analyses. Curr Protoc Bioinformatics. 2016;54(1). 10.1002/cpbi.5 27322403

[34] Barrett T, Wilhite SE, Ledoux P (2013). NCBI GEO: archive for functional genomics data sets--update. Nucleic Acids Res.

[35] Piñero J, Ramírez-Anguita JM, Saüch-Pitarch J (2020). The DisGeNET knowledge platform for disease genomics: 2019 update. Nucleic Acids Res.

[36] Amberger JS, Bocchini CA, Schiettecatte F, Scott AF, Hamosh A (2015). OMIM.org: online Mendelian inheritance in man (OMIM®), an online catalog of human genes and genetic disorders. Nucleic Acids Res.

[37] Wickham H, Ggplot2 (2016). Elegant graphics for data analysis.

[38] Chen H, Boutros PC (2011). VennDiagram: a package for the generation of highly-customizable Venn and Euler diagrams in R. BMC Bioinformatics.

[39] Szklarczyk D, Gable AL, Lyon D (2019). STRING v11: protein-protein association networks with increased coverage, supporting functional discovery in genome-wide experimental datasets. Nucleic Acids Res.

[40] Gu Z, Gu L, Eils R, Schlesner M, Brors B (2014). Circlize implements and enhances circular visualization in R. Bioinformatics..

[41] R Core Team. R: A language and environment for statistical computing. R Foundation for statistical Computing; 2020.

[42] Shannon P, Markiel A, Ozier O (2003). Cytoscape: a software environment for integrated models of biomolecular interaction networks. Genome Res.

[43] Yu G, Wang LG, Han Y, He QY (2012). clusterProfiler: an R package for comparing biological themes among gene clusters. OMICS..

[44] Pathview: an R/Bioconductor package for pathway-based data integration and visualization | Bioinformatics | Oxford Academic. 10.1093/bioinformatics/btt285 PMC3702256 23740750

[45] Trott O, Olson AJ (2010). AutoDock Vina: improving the speed and accuracy of docking with a new scoring function, efficient optimization, and multithreading. J Comput Chem.

[46] Laskowski RA, Swindells MB (2011). LigPlot+: multiple ligand-protein interaction diagrams for drug discovery. J Chem Inf Model.

[47] Konno H, Suzuki H, Tadakuma T (1987). Antitumor effect of adriamycin entrapped in liposomes conjugated with anti-human alpha-fetoprotein monoclonal antibody. Cancer Res.

[48] Benonisson H, Altıntaş I, Sluijter M (2019). CD3-bispecific antibody therapy turns solid tumors into inflammatory sites but does not install protective memory. Mol Cancer Ther.

[49] Kanehisa M, Goto S (2000). KEGG: Kyoto encyclopedia of genes and genomes. Nucleic Acids Res.

[50] Kanehisa M (2019). Toward understanding the origin and evolution of cellular organisms. Protein Sci.

[51] Kanehisa M, Furumichi M, Sato Y, Ishiguro-Watanabe M, Tanabe M (2021). KEGG: integrating viruses and cellular organisms. Nucleic Acids Res.

[52] Munck JM, Berdini V, Bevan L (2021). ASTX029, a novel dual-mechanism ERK inhibitor, modulates both the phosphorylation and catalytic activity of ERK. Mol Cancer Ther.

[53] Juskevicius D, Lorber T, Gsponer J (2016). Distinct genetic evolution patterns of relapsing diffuse large B-cell lymphoma revealed by genome-wide copy number aberration and targeted sequencing analysis. Leukemia..

[54] van der Woude LL, Gorris MAJ, Halilovic A, Figdor CG, de Vries IJM (2017). Migrating into the tumor: a roadmap for T cells. Trends Cancer.

[55] Leivonen SK, Pollari M, Brück O (2019). T-cell inflamed tumor microenvironment predicts favorable prognosis in primary testicular lymphoma. Haematologica..

[56] Scott DW, Gascoyne RD (2014). The tumour microenvironment in B cell lymphomas. Nat Rev Cancer.

[57] Abès R, Gélizé E, Fridman WH, Teillaud JL (2010). Long-lasting antitumor protection by anti-CD20 antibody through cellular immune response. Blood..

[58] Hilchey SP, Hyrien O, Mosmann TR (2009). Rituximab immunotherapy results in the induction of a lymphoma idiotype-specific T-cell response in patients with follicular lymphoma: support for a “vaccinal effect” of rituximab. Blood..

[59] Murakami W, Tozaki M, Sasaki M (2020). Correlation between 18F-FDG uptake on PET/MRI and the level of tumor-infiltrating lymphocytes (TILs) in triple-negative and HER2-positive breast cancer. Eur J Radiol.

[60] Wang Y, Zhao N, Wu Z (2020). New insight on the correlation of metabolic status on 18F-FDG PET/CT with immune marker expression in patients with non-small cell lung cancer. Eur J Nucl Med Mol Imaging.

[61] Reinfeld BI, Madden MZ, Wolf MM (2021). Cell-programmed nutrient partitioning in the tumour microenvironment. Nature..

[62] Zheng Z, Sun R, Zhao HJ (2019). MiR155 sensitized B-lymphoma cells to anti-PD-L1 antibody via PD-1/PD-L1-mediated lymphoma cell interaction with CD8+T cells. Mol Cancer.

[63] Freeman GJ, Sharpe AH, Kuchroo VK (2002). Protect the killer: CTLs need defenses against the tumor. Nat Med.

[64] Palucka AK, Coussens LM (2016). The basis of Oncoimmunology. Cell..

[65] Mullins SR, Vasilakos JP, Deschler K (2019). Intratumoral immunotherapy with TLR7/8 agonist MEDI9197 modulates the tumor microenvironment leading to enhanced activity when combined with other immunotherapies. J Immunother Cancer.

[66] Huang L, Xu H, Peng G (2018). TLR-mediated metabolic reprogramming in the tumor microenvironment: potential novel strategies for cancer immunotherapy. Cell Mol Immunol.

[67] Tanaka T, Narazaki M, Kishimoto T (2014). IL-6 in inflammation, immunity, and disease. Cold Spring Harb Perspect Biol.

[68] Wu T, Dai Y (2017). Tumor microenvironment and therapeutic response. Cancer Lett.

[69] Muthuswamy R, Corman JM, Dahl K, Chatta GS, Kalinski P (2016). Functional reprogramming of human prostate cancer to promote local attraction of effector CD8(+) T cells. Prostate..

[70] Harlin H, Meng Y, Peterson AC (2009). Chemokine expression in melanoma metastases associated with CD8+ T-cell recruitment. Cancer Res.

[71] Andersson A, Yang SC, Huang M (2009). IL-7 promotes CXCR3 ligand-dependent T cell antitumor reactivity in lung cancer. J Immunol.

[72] Berghuis D, Santos SJ, Baelde HJ (2011). Pro-inflammatory chemokine-chemokine receptor interactions within the Ewing sarcoma microenvironment determine CD8(+) T-lymphocyte infiltration and affect tumour progression. J Pathol.

[73] Slaney CY, Kershaw MH, Darcy PK (2014). Trafficking of T cells into tumors. Cancer Res.

[74] Mauldin IS, Wang E, Deacon DH, Olson WC, Bao Y, Slingluff CL (2015). TLR2/6 agonists and interferon-gamma induce human melanoma cells to produce CXCL10. Int J Cancer.

[75] Hargadon KM (2016). Dysregulation of TGFβ1 activity in Cancer and its influence on the quality of anti-tumor immunity. J Clin Med.

[76] Weber R, Groth C, Lasser S (2021). IL-6 as a major regulator of MDSC activity and possible target for cancer immunotherapy. Cell Immunol.

[77] Fraietta JA, Lacey SF, Orlando EJ (2018). Determinants of response and resistance to CD19 chimeric antigen receptor (CAR) T cell therapy of chronic lymphocytic leukemia. Nat Med.

