# Supplementary material for: Qin Huang formula enhances the effect of Adriamycin in B-cell lymphoma via increasing tumor infiltrating lymphocytes by targeting toll-like receptor signaling pathway
Source: BMC Complement Med Ther. 2022 Jul 11;22:185. doi: 10.1186/s12906-022-03660-8 (PMC9272877; doi:10.1186/s12906-022-03660-8)
Supplement: Supplementary file 1 — Additional file 1: Figure S1. Pre-study data of the effective dosage of QHF. A.The image of the tumor blocks of the three groups. B.Thebodyweight of the mice in three groups during the experiment. C. The tumor blocks weight of the three groups. Compared with the LD group (n=5), the tumor weight was significantly decreased in the HD group (n=5, **p<0.01). Compared with the MD group (n=5), the tumor weight was remarkably lowered in the HD group (n=5, *p<0.05) [file 12906_2022_3660_MOESM1_ESM.pdf]

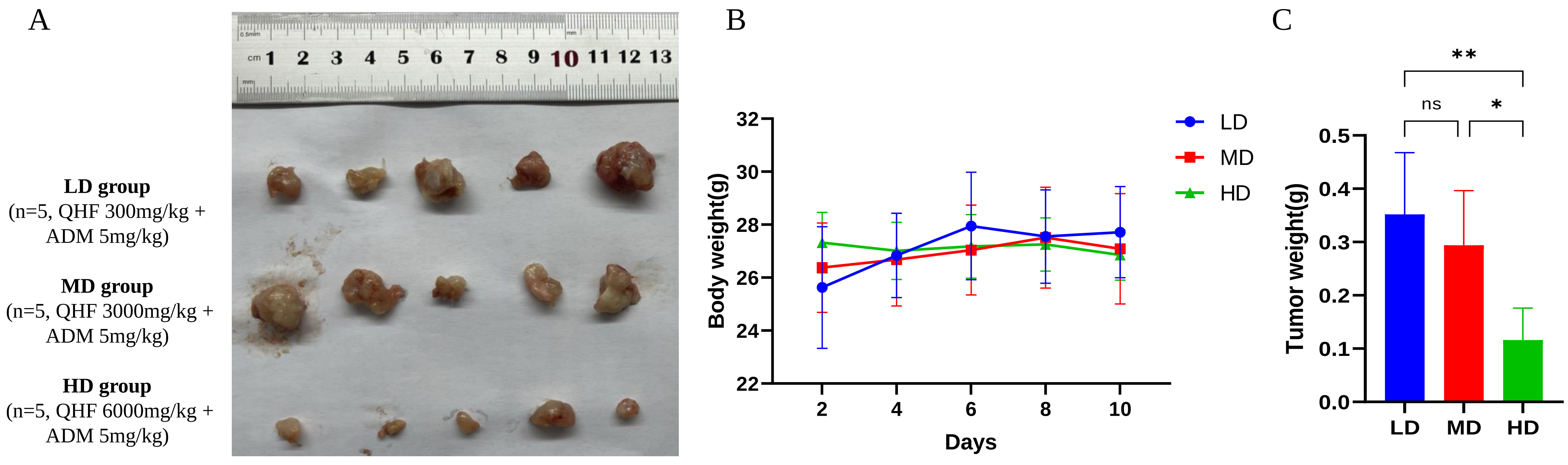

Figure S1. Pre-study data of the effective dosage of QHF. A. The image of the tumor blocks of the three groups. B. The body weight of the mice in three groups during the experiment. C. The tumor blocks weight of the three groups. Compared with the LD group (n=5), the tumor weight was significantly decreased in the HD group (n=5, **$p<0.01$). Compared with the MD group (n=5), the tumor weight was remarkably lowered in the HD group (n=5, *$p<0.05$).
