# Supplementary material for: Qin Huang formula enhances the effect of Adriamycin in B-cell lymphoma via increasing tumor infiltrating lymphocytes by targeting toll-like receptor signaling pathway
Source: BMC Complement Med Ther. 2022 Jul 11;22:185. doi: 10.1186/s12906-022-03660-8 (PMC9272877; doi:10.1186/s12906-022-03660-8)
Supplement: Supplementary file 4 — Additional file 4: Figure S2. IHC stainingsfor M1 and M2 macrophages. A. Representative images of IHC staining for M1 macrophages among the three groups (200×). B. Bar plot of the positive cell rate of M1 macrophages of the control group (n=3), ADM group (n=3), and QHF+ADM group (n=3). C. Representative images of IHC staining for M2 macrophages among the three groups (200×). D. Bar plot of the positive cell rate of M2 macrophages of the control group (n=3), ADM group (n=3), and QHF+ADM group (n=3).Ns: p>0.05 [file 12906_2022_3660_MOESM4_ESM.pdf]

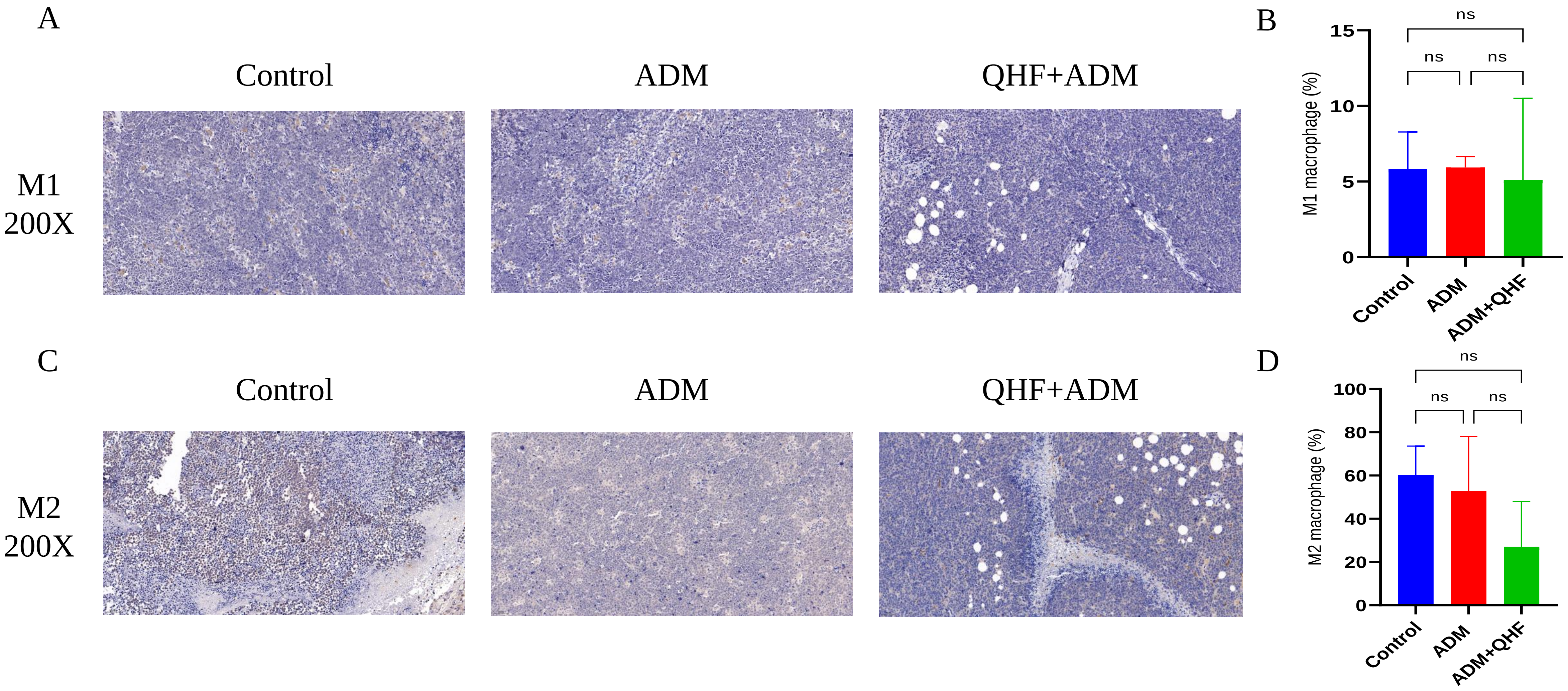

Figure S2. IHC stainings for M1 and M2 macrophages. A. Representative images of IHC staining for M1 macrophages among the three groups (200×). B. Bar plot of the positive cell rate of M1 macrophages of the control group (n=3), ADM group (n=3), and QHF+ADM group (n=3). C. Representative images of IHC staining for M2 macrophages among the three groups (200×). D. Bar plot of the positive cell rate of M2 macrophages of the control group (n=3), ADM group (n=3), and QHF+ADM group (n=3). Ns: $p>0.05$.
