# Supplementary material for: Qin Huang formula enhances the effect of Adriamycin in B-cell lymphoma via increasing tumor infiltrating lymphocytes by targeting toll-like receptor signaling pathway
Source: BMC Complement Med Ther. 2022 Jul 11;22:185. doi: 10.1186/s12906-022-03660-8 (PMC9272877; doi:10.1186/s12906-022-03660-8)
Supplement: Supplementary file 5 — Additional file 5: Figure S3. ELISA results of IL-2 and IL-17. A. Concentration of IL-2 of the control group (n=3), ADM group (n=3), and ADM+QHF group (n=3). There is no significant difference between the three groups (p>0.05). B. Concentration of IL-17 of the control group (n=3), ADM group (n=3), and ADM+QHF group (n=3). There is no significant difference between the three groups (p>0.05) [file 12906_2022_3660_MOESM5_ESM.pdf]

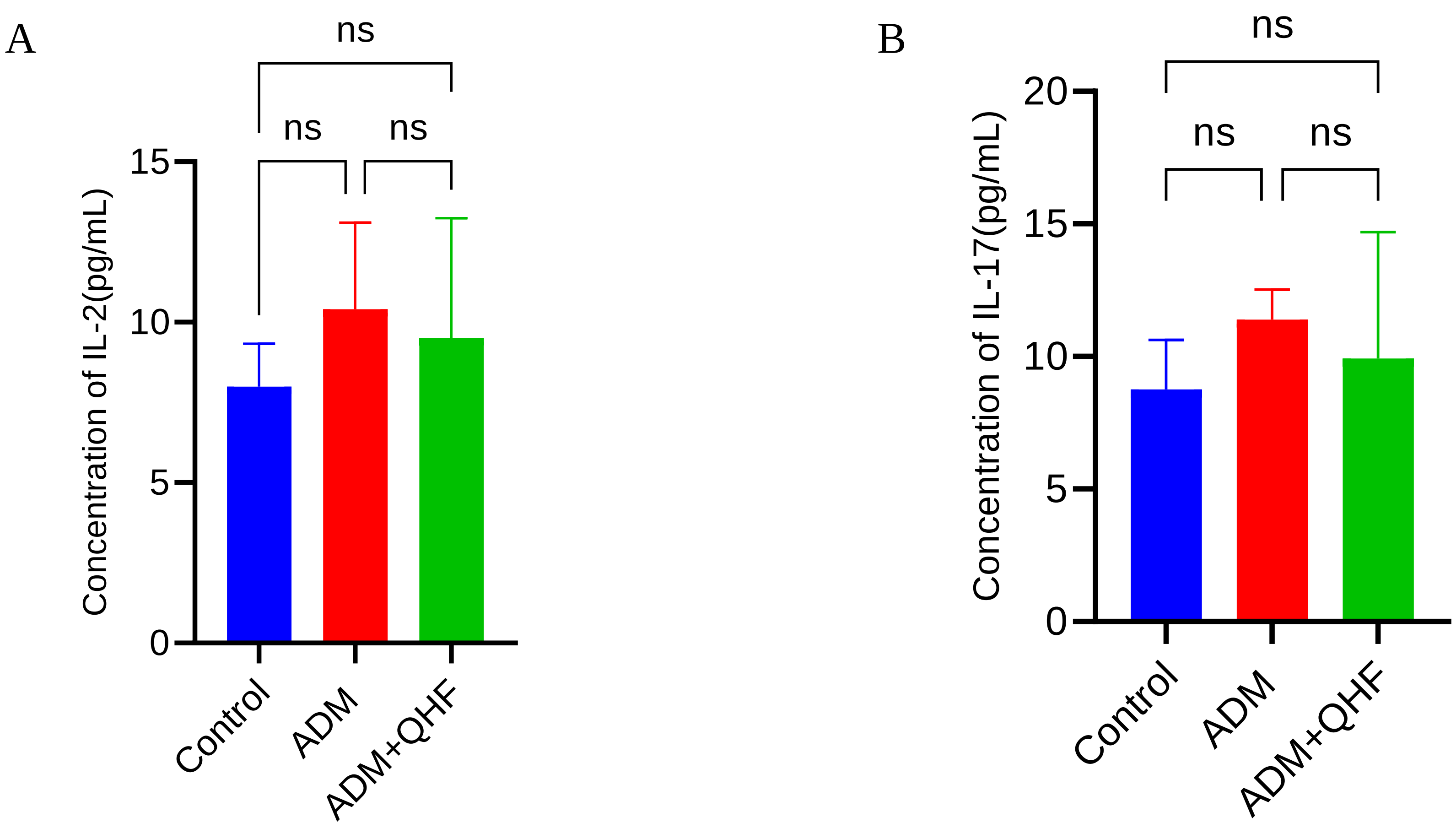

Figure S3. ELISA results of IL-2 and IL-17. A. Concentration of IL-2 of the control group (n=3), ADM group (n=3), and ADM+QHF group (n=3). There is no significant difference between the three groups ($p>0.05$). B. Concentration of IL-17 of the control group (n=3), ADM group (n=3), and ADM+QHF group (n=3). There is no significant difference between the three groups ($p>0.05$)
