# Supplementary material for: Qin Huang formula enhances the effect of Adriamycin in B-cell lymphoma via increasing tumor infiltrating lymphocytes by targeting toll-like receptor signaling pathway
Source: BMC Complement Med Ther. 2022 Jul 11;22:185. doi: 10.1186/s12906-022-03660-8 (PMC9272877; doi:10.1186/s12906-022-03660-8)
Supplement: Supplementary file 6 — Additional file 6: Figure S4. Image J results of the WB bands. A. Bar plot of the expression of TLR2. Compared with the Control group (n=3) and the ADM group (n=3), the ADM+QHF group (n=3) significantly increased the expressions of TLR2 (v.s. Control group, ***p<0.001; v.s. ADM group, ****p<0.0001). B. Bar plot of the expression of p38.Compared with the Control group (n=3), the level of p38 MAPK was remarkably increased in ADM group (n=3, ***p<0.001) and ADM+QHF group (n=3, ***p<0.001) [file 12906_2022_3660_MOESM6_ESM.pdf]

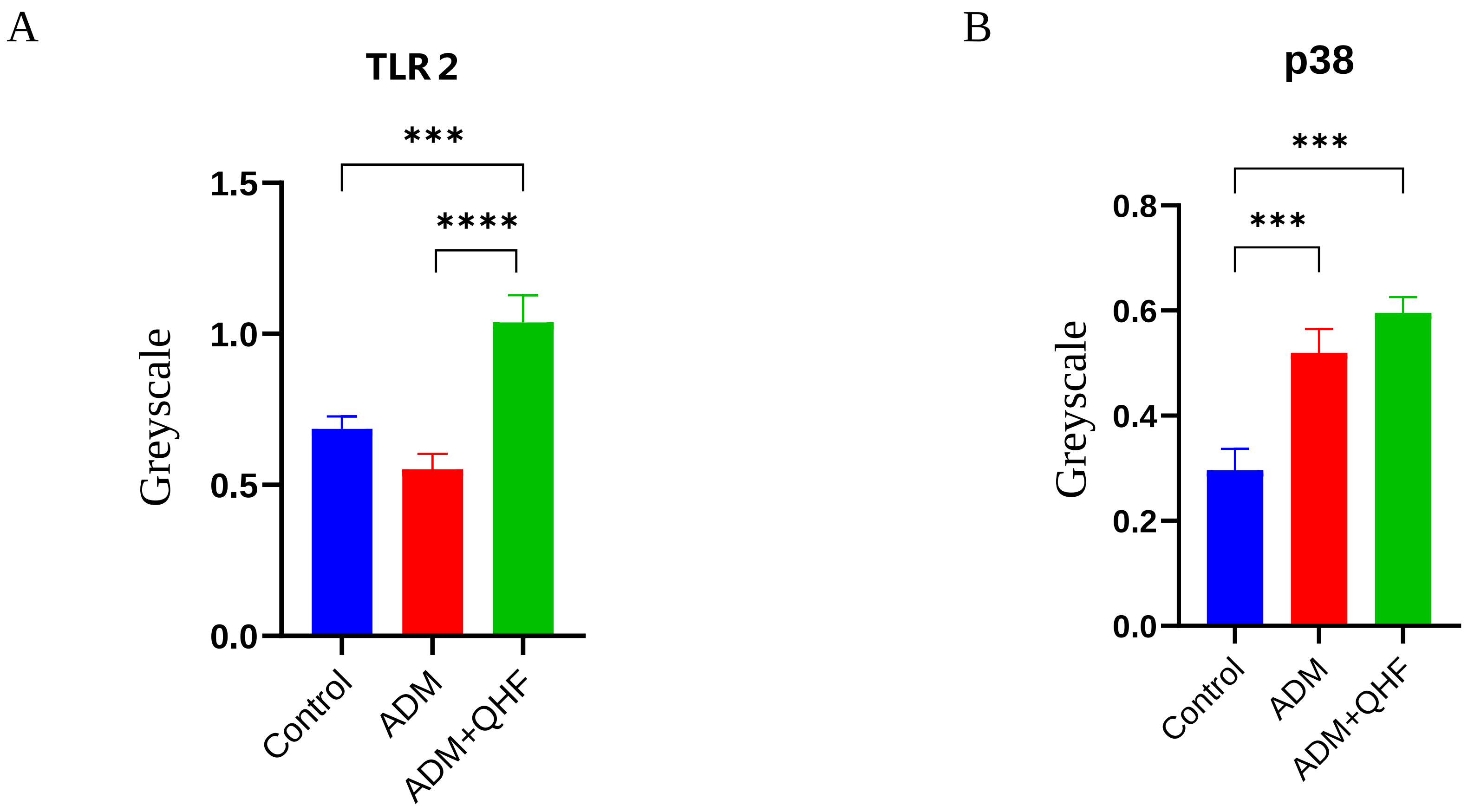

Figure S4. Image J results of the WB bands. A. Bar plot of the expression of TLR2. Compared with the Control group (n=3) and the ADM group (n=3), the ADM+QHF group (n=3) significantly increased the expressions of TLR2 (v.s. Control group, ***p<0.001; v.s. ADM group, ****p<0.0001). B. Bar plot of the expression of p38. Compared with the Control group (n=3), the level of p38 MAPK was remarkably increased in ADM group (n=3, ***p<0.001) and ADM+QHF group (n=3, ***p<0.001).
